# How to facilitate employees’ green behavior? The joint role of green human resource management practice and green transformational leadership

**DOI:** 10.3389/fpsyg.2022.906869

**Published:** 2022-08-11

**Authors:** Tingting Chen, Zhanyong Wu

**Affiliations:** ^1^School of Economics and Trade, Henan Polytechnic Institute, Nanyang, China; ^2^Fanli Business School, Nanyang Institute of Technology, Nanyang, China

**Keywords:** green human resource management, green transformational leadership, green behavior, green mindfulness, green self-efficacy

## Abstract

Given the severity of today’s environmental issues, companies are increasingly making green concepts a key component of their operational strategies. As an essential complement to corporate environmental strategy, employees’ green behavior has received attention from all sectors of society. Based on resource conservation theory, this study explores the formation mechanism of employees’ green behaviors in enterprises starting from two green management tools: green human resource management (HRM) practices and green transformational leadership. Through two-stage questionnaire research, 296 sample data points were obtained, and the research hypotheses were tested by using linear regression analysis. The results showed that green HRM practices in enterprises enhance employees’ green mindfulness and thus stimulate their green behaviors and that green transformational leadership and green self-efficacy play a positive moderating role in the above relationship. These results support the applicability of resource conservation theory in green management and suggest that green HRM practices and green transformational leadership can be used together in the process of green management.

## Introduction

Employees’ green behavior can be generally understood as employee activities that aim to conserve natural resources and the ecological environment and that work to reduce environmental degradation and improve environmental quality (Norton et al., 2015). Given the critical value of employees’ green behavior, scholars have begun to explore what management measures organizations can use to motivate employees to pursue green practices. Current scholarly research on corporate employees’ green behaviors has focused on individual traits (Tariq et al., 2020), leadership traits (Wang et al., 2018), and organizational climate (Zientara and Zamojska, 2018). In particular, green transformational leadership (Tian and Jiang, 2021) and green human resource management (HRM) practice (Renwick et al., 2016; Shen et al., 2018; Hazarika and Zhang, 2019; Fawehinmi et al., 2020; Farooq et al., 2021) have attracted much attention as two sharp tools of green management.

As a soft means of green management, green transformational leadership aims to convey the “subjective” norms of protecting the environment to organization members to stimulate green behavior among subordinates (Robertson and Barling, 2013). As a hard means of green management, green human resource management practice regulates employees’ green behavior at the system and policy levels (Dumont et al., 2017). However, previous studies have discussed the effects of green transformational leadership and green human resource management practices in a fragmented way, ignoring the joint mechanism of the two on employees’ green behavior (Robertson and Barling, 2013; Dumont et al., 2017; Tian and Robertson, 2019; Yusliza et al., 2020). Theoretically, unilateral factors have difficulty explaining most of the variations in green behavior and are not conducive to a comprehensive and systematic understanding of the inducing influences on employees’ green behavior. As Leroy et al. (2018) pointed out, leadership behavior and human resource management practice have a common goal of managing people more effectively in the organization. Based on this commonality, they can coexist in the organization and shape employees’ workplace behavior together. Therefore, considering the interaction between the two can not only improve the research’s explanatory power but also help reveal how multiple situational factors shape employees’ green behavior. Following this logic, this study aims to explore the interaction between green transformational leadership and green human resource management practice on employees’ green behavior and its internal mechanisms, covering the gap in corporate environmental responsibility fulfillment research at the micro-level.

According to resource conservation theory (Hobfoll, 1989), when individuals have more resources, they are more willing to engage in resource investment behavior. Green human resource management practices, such as green training and green empowerment, are typical work resources that improve employees’ green mindfulness to a certain extent (Chen et al., 2015; Ren et al., 2018), which helps employees show more green behaviors. At the same time, the effectiveness of HRM practices depends on organizational leadership to a certain extent. The leadership characteristics of green transformational leadership can effectively coordinate the relationship between organizational strategy and human resource management, enhance employees’ green mindfulness, and then influence green behavior. Revealing the intermediary role of green mindfulness can provide a more specific explanation for the interactive effect of the two green management tools. In addition, the difference in individual self-efficacy may lead to different degrees of green behavior among employees under the same human resource management. Therefore, if enterprises want to motivate employees to show attitudes and behaviors that conform to the development of organizational strategy and realize the maxim, “love what the company loves and do what the company does,” on environmental issues, they must jointly apply green transformational leadership and green HRM practice and then shape employees’ green mindfulness and influence employees’ green behavior.

In summary, this study will integrate green transformational leadership (soft means) and green HRM practice (hard system) and explore how they affect employees’ green behavior together. The main purposes of this study are as follows: first, to explore the interaction effect of green HRM practice and green transformational leadership on employees’ green behavior; second, to test the intermediary role of green mindfulness; and finally, to test the regulatory effect of green self-efficacy.

## Theoretical background and hypothesis development

### Hard measures to stimulate employees’ green behavior: Green human resource management

Existing research suggests that HRM practices positively influence employees’ attitudes, motivations, and behaviors and that employees’ perceptions of HRM are significant predictors of their work attitudes and behaviors (Tian and Jiang, 2021). Green HRM practices are mandatory systems to support green management that aim to integrate “environmental issues” into HRM functional modules (Renwick et al., 2013; Yong et al., 2020) and regulate employees’ green behaviors at the institutional level. According to Renwick et al. (2013), green HRM practices are mainly manifested in introducing green issues into recruitment, training, performance evaluation, compensation and benefits management activities. Employees are more likely to demonstrate green behaviors through these green-oriented management activities. The specific reasons are as follows. First, by recruiting green-oriented staff and doing green practices an employer brand, companies can attract and bring in environmentally conscious employees, and these employees will naturally show green behavior after entering the company. Second, by organizing environmental skills training, companies can enrich employees’ environmental knowledge and train them to solve environmental problems so that employees are more capable of implementing green behavior. Third, by introducing green issues to employees, companies can make employees more likely to show green behavior. Fourth, by incorporating employees’ green behavior into the performance evaluation and compensation system, employees can clarify their responsibilities and realize that they can earn rewards for being more active in environmentally protective behavior (Dumont et al., 2017). In conclusion, when employees experience green HRM practices, they will show more green behaviors.

*H1*: There is a positive correlation between employees’ perceived green HRM practices and green behavior.

### Soft measures to stimulate green behavior: Green transformational leadership

Green transformational leadership refers to leaders motivating employees to achieve green goals and encouraging employees to demonstrate green behaviors beyond the expected level (Chen and Chang, 2013; Peng et al., 2019a), including through influence, motivation, intellectual stimulation and personalized care (Robertson, 2018). Green influence refers to leaders acting as environmental role models and influencing the green behavior of subordinates through their charisma. Green motivation means that leaders encourage employees to go beyond their short-term self-interest and work hard to achieve green goals. Green intellectual stimulation refers to leaders inspiring employees to challenge old ideas and use new methods to solve environmental problems. Green personalized care refers to leaders who value their employees’ green contributions and help them develop environmental skills (Robertson, 2018). Through the green influence and green motivation, green transformational leaders help employees understand the importance and feasibility of green behaviors to realize that green behaviors are encouraged and expected by organizations and that they should demonstrate green behaviors (Robertson and Barling, 2013). At the same time, through green intellectual stimulation and green personalized care, green transformational leaders can develop employees’ ability to think about environmental issues, enrich their environmental knowledge, and help them develop environmental skills so that they have the will and ability to demonstrate green behaviors (Kura, 2016). In summary, when employees perceive that leaders show green transformational leadership behavior, they will show more green behavior.

*H2*: There is a positive correlation between employees’ perception of green transformational leadership and green behavior.

### The interaction of green human resource management practice and green transformational leadership on employee green behavior

The effectiveness of green HRM practices also depends to some extent on the organization’s leadership, and motivating employees to engage in green behaviors through leaders is essential for companies to establish a green image. In this study, green transformational leadership and green human resource management practices are two important reference sources before employees take green actions. When employees perceive a high level of green human resource management practice, and the more leaders show a high level of green transformational leadership behavior, the more likely employees are to perceive that both sides are consistent, which can accelerate the recognition and adoption of green ideas, green expectations and green behavior. Specifically, when the organization insists on green orientation in human resource management activities, employees will realize the importance and value of green management. In this case, as long as the leader continues to show a high level of green transformational leadership behavior (Robertson and Barling, 2013), employees can obtain more consistent clues from their work environment, more firmly believe in the importance of green behavior, and believe that green human resource management practices can be implemented within the department. This is conducive to strengthening employees’ determination to engage in green behavior.

However, when employees feel that there is a difference between green transformational leadership and the green human resource management level, employees will receive two inconsistent messages. Because the two messages have a certain weight in the organization, employees will be uncertain and initiate a cognitive processing process dominated by negative prejudice (Roeck and Farooq, 2018), that is, pay more attention to the clues that will bring them losses or negative impacts. For example, under the practice of low-level green human resource management, the higher the degree of green transformational leadership perceived by employees, the more likely they are to believe that engaging in green behavior only satisfies the personal wishes of leaders and benefits them personally but will not be recognized and rewarded by the formal system of the organization. In this case, employees will regard “engaging in green behavior” as a loss of time or energy that cannot obtain benefits from the organization, which will consequently fail to increase the probability of green behavior. In summary, this study proposes the following hypothesis.

*H3*: Employees’ perceived green transformational leadership and green human resource management practice positively interact with employees’ green behavior. Under a high degree of green human resource management practice, green transformational leadership strongly impacts employees’ green behavior.

### The mediating role of the green mindfulness of corporate employees

Mindfulness refers to a state of conscious awareness, living in the present moment, and nonjudgment (Barbaro and Pickett, 2016). Scholars in the field of organizational management define mindfulness as “a receptive attention and awareness of current events and experiences” (Peng et al., 2019b). Research has found that mindfulness in the workplace positively impacts employees’ work well-being, pro-environmental behavior, and learning (Liu and Li, 2020). Chen et al. (2015) define green mindfulness as a type of awareness in which individuals focus their attention on the various contexts and contents of environmental well-being in the present. Green mindfulness includes four characteristics: sensitivity to the environment, openness to new information, multiperspective cognition, and awareness and attention to different backgrounds (Dharmesti et al., 2020). Many researchers have recognized that HRM practices usually do not directly influence employees’ work attitudes and behaviors; rather, this influence is transmitted through specific social and psychological processes (Tian and Jiang, 2021). According to resource conservation theory (Hobfoll, 1989), multiple resources in green HRM systems (e.g., green training, green job design) contribute to increasing employees’ psychological resources, such as green mindfulness, green sensitivity, and green attention (Guerci et al., 2016; Liu and Li, 2020). Among them, green mindfulness, as a positive psychological resource, is an essential driver for employees to engage in green behaviors. Employees with green mindfulness are concerned about the environmental consequences of their actions and are more likely to participate in implementing and accepting managers’ strategic decisions regarding corporate environmental responsibility (Liu and Li, 2020), which in turn directly affects employees’ green behaviors in the workplace.

Green Transformational Leadership (Chen and Chang, 2013) and Green Human Resource Management Practice (Dumont et al., 2017) are two important information sources for employees to understand and understand environmental protection goals, which can interactively influence employees’ green beliefs. This interaction is shown as follows: Green transformational leadership can strengthen the influence of green human resource management practice on employees’ green beliefs. Specifically, under green transformational leadership, employees will have goals and beliefs about environmental practices communicated to them (Robertson, 2018). At this time, if employees experience high-intensity green human resource management practices, they can perceive that the organization attaches great importance to environmental protection issues and their environmental protection responsibilities (Tian and Jiang, 2021) from a series of systems, such as green-oriented training, performance management and salary and welfare (Dumont et al., 2017), thus resulting in more green behaviors.

*H4*: The interaction between green human resource management practice and green transformational leadership positively affects employees’ green behavior through green mindfulness.

### Moderating effect of green self-efficacy

Green self-efficacy is a concept of self-efficacy that incorporates green environmental factors, which refers to individuals’ evaluation of their ability to perform different activities to achieve green goals (Chen et al., 2015; Guo et al., 2019). Individuals with high levels of green self-efficacy believe they have the ability and confidence to accomplish specific green tasks. Previous studies have found that green self-efficacy, as a self-perception, positively impacts green behavior (Huang, 2016). According to resource conservation theory (Hobfoll, 2001), employees with high green self-efficacy usually have abundant psychological resources (e.g., positive thoughts, confidence, courage) and believe that they are capable of completing challenging tasks (Liu and Li, 2020). In this case, employees will try to obtain more resources from the green HRM system (e.g., green empowerment) and use green behavioral opportunities to create resource surpluses. Therefore, employees with high green self-efficacy will understand the intention and purpose of green HRM practices that are positively implemented by the organization and then make full use of the available internal and external resources to actively participate in green activities; in this way, the impact of green HRM is enhanced. In contrast, when employees’ green self-efficacy is at a low level, even if the organization provides employees with various resources (e.g., green training opportunities, green rewards), employees’ sense of obligation to engage in green activities will not be strengthened due to their lack of positive intention to participate in green activities; that is, the influence of green HRM will be weakened. In summary, this paper proposes the following hypothesis.

*H5*: Employees’ green self-efficacy plays a positive moderating role in the relationship between green mindfulness and green behavior.

**Figure 1:**
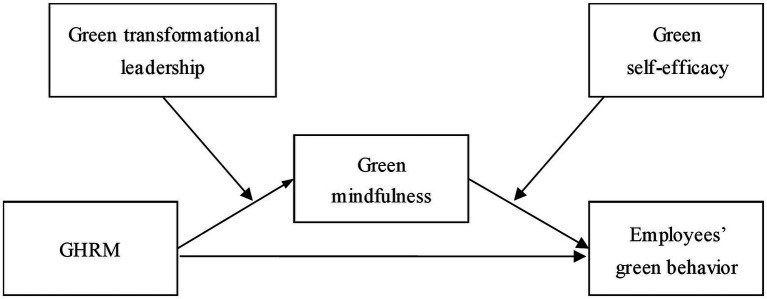
The theoretical model of the green behavior of Chinese employees.

In summary, the employee green behavior model is constructed in the context of Chinese culture, as shown in Figure 1. To test these hypotheses, we carry out a survey that is presented in the next part of this paper.

## Research design

### Sample and data collection

In recent years, the Chinese government has introduced many environmental regulations urging companies to shift to an environmentally sustainable development model. This study argues that enterprises’ adoption of green HRM practices is conducive to the construction of a differentiated competitive advantage. Green employee behavior complements organizational policies and helps promote corporate green management. The samples of existing empirical studies on green HRM are mainly from ISO14001-certified companies, which usually implement green HRM practices (Shen et al., 2018). Given this, this study selected four ISO14001-certified companies representing the tourism, manufacturing, construction, and pharmaceutical industries. Before the formal survey, the researchers contacted the individuals concerned by phone and email to explain the purpose of the study and how the survey would be conducted. After obtaining their consent, the researchers proceeded to the formal survey process. A combination of an on-site survey and commissioned method was used to collect data. To avoid homogeneous variance, this study used two-time points to collect data. Four hundred thirty questionnaires were distributed to employees from July to September 2020. Employees answered questions related to demographic variables, jobs, green HRM, and green mindfulness; 387 questionnaires were returned, and 358 valid questionnaires were obtained after eliminating invalid questionnaires. Three hundred fifty-eight questionnaires addressing green transformational leadership, self-efficacy, and behavior were distributed again in November–December 2020 to 358 employees. Three hundred twenty-five questionnaires were returned, of which 296 questionnaires were valid, with a good return rate of 91.08%. The characteristics of the valid sample are shown in Table 1. The respondents have a wide range of characteristic attributes and coverage that are suitable for further empirical analysis.

**Table 1:** Descriptive statistics of samples.

Demographic information		Respondents (*N* = 296)	Percentage (%)
Gender	Male	167	56.42
	Female	129	43.58
Age	Less than 30 years	125	42.23
	31–40 years	113	38.18
	41–50 years	41	13.85
	51 and over	17	5.74
Education	High school and above	57	19.26
	Associate degree	93	31.42
	Bachelor’s degree	114	38.51
	Master’s degree or above	32	10.81
Organizational Tenure	1–3 years	136	45.95
	4–6 years	78	26.35
	7–9 years	44	14.86
	10 years and above	38	12.84
Position	Production	114	38.51
	Administrative	67	22.64
	Technical	55	18.58
	R&D	20	6.76
	Other	40	13.51

### Measurement of variables

All the scales in this study were obtained from foreign literature, and a two-way translation procedure was used to translate the main variable scales into Chinese. All items were scored on a five-point Likert scale, with “1” indicating “strongly disagree” and “5” indicating “strongly agree.”

#### Green HRM

Since the improvement of HRM systems on organizational performance is based on employees’ perceptions of HRM practices, most studies use employees’ perceptions of green HRM practices to measure green HRM (Dumont et al., 2017; Shen et al., 2018). This study uses the Green HRM scale developed by Dumont et al. (2017) with six questions, such as “My company has set green appraisal goals for its employees.” In this study, the Cronbach’s alpha coefficient of this scale was 0.903, and the CR value was 0.868.

#### Green mindfulness

The six-question green mindfulness scale developed by Chen et al. (2014) was used, with example questions such as “Employees are free to discuss environmental problems and issues.” In this study, the Cronbach’s alpha coefficient of the scale was 0.823, and the CR value was 0.821.

#### Green transformational leadership

Green transformational leadership was measured using the scale developed by Mittal and Dhar (2016). Items that were repetitive, ambiguous, and did not fit the Chinese context were removed, resulting in questions such as “My leader motivates me to work in an environmentally friendly way,” and “My leader states that he is committed to improving the environmental performance of the organization,” and “My leader states many times that he values the natural environment.” The Cronbach’s alpha coefficient for the scale was 0.921, and the CR value was 0.929.

#### Green self-efficacy

The six-item green self-efficacy scale developed by Chen et al. (2014) was used, with questions such as “I believe I am capable of taking measures to mitigate global warming and stop climate change,” “I can think of some environmental ideas,” “I feel that I can accomplish most of my environmental goals,” and “I feel that I am capable and efficient in handling the tasks assigned by my supervisor to accomplish the environmental goals of my company.” The Cronbach’s alpha coefficient for this scale in this study was 0.900, and the CR value was 0.893.

#### Green behaviors

The scale developed by Kim et al. (2016) was used to measure employees’ green behaviors. The scale was adjusted appropriately according to the content of this study without changing the meaning of the scale. The final results included “I turn off the lights when I leave an unoccupied room,” “I sort the garbage and recycle garbage at work,” and “I am very concerned about water waste.” The Cronbach’s alpha coefficient for the scale was 0.890, and the CR value was 0.888.

#### Control variables

According to previous studies (Dumont et al., 2017), age, gender, education, years of experience, and position were used as control variables in this study.

## Results

### Exploratory factor analysis

As reported above, the range of Cronbach’s α coefficients was 0.823 ~ 0.921; therefore, the reliability of each scale in this study was good, and all had high internal consistency. By using principal component analysis for pivoting and extracting common factors with eigenvalues greater than 1, the results showed that five common elements could be removed, and the cumulative explained variance of the extracted common factors reached 68.319%. The above results indicated that the scales of this study could accurately measure the content of the study. To verify whether there was common method bias, this study conducted Harman’s one-way test on the collected data by referring to Podsakoff et al. (2003) test for common method bias and performed exploratory factor analysis by putting all the measured items of the variables together. The cumulative sum of squares of the first ranked extracted loadings was 34.148%, which did not exceed 40% of the required range, so the collected questionnaire data did not have homogeneity. To test the suitability of the sample for exploratory factor analysis, the KMO value and Bartlett’s spherical test were calculated using SPSS 23.0 software. The results showed that the KMO value of the sample was 0.888, and Bartlett’s spherical test was significant (*p* = 0.000), indicating that the sample data were suitable for exploratory factor analysis.

### Confirmatory factor analysis

In this study, validated factor analysis was performed on the data using Amos 24.0 software to test the discriminant validity of the variables by observing the fitted values of the models. As seen in Table 2, the one-factor model had the worst fit (RMSEA = 0.112, CFI = 0.789, TLI = 0.761), and the five-factor model had the best fit index relative to the other models (RMSEA = 0.054, CFI = 0.953, TLI = 0.944) and reached an acceptable level, indicating that the five variables in this study had good discriminant validity.

**Table 2:** Confirmatory factor analysis (*N* = 296).

*Model*	χ^2^	*df*	χ^2^*/df*	*IFI*	*TLI*	*CFI*	*RMSEA*
Five-factor model	730.687	391	1.869	0.954	0.944	0.953	0.054
Four-factor model	953.110	406	2.348	0.925	0.914	0.925	0.068
Three-factor model	1391.300	408	3.410	0.866	0.846	0.865	0.090
Two-factor model	1855.748	410	4.526	0.802	0.774	0.801	0.109
Single-factor model	1943.748	411	4.729	0.790	0.761	0.789	0.112

Five-factor model: GHRM, GM, GTL, GSE, GB; Four-factor model: GHRM + GTL, GM, GSE, GB; Three-factor model: GHRM + GTL, GM + GSE, GB; Two-factor model: GHRM, GM + GTL + GSE + GB; One-factor model: GHRM + GM + GTL + GSE + GB.

### Descriptive statistics and correlation analysis

Table 3 shows the means, standard deviations and correlations between the variables involved in this study.

**Table 3:** Mean value, standard deviation and correlation coefficient of each variable (*N* = 296).

Variable	Mean	SD	1	2	3	4	
GHRM	3.421	0.444	0.735				
GM	3.292	0.345	0.639^**^	0.674			
GB	3.328	0.396	0.608^**^	0.677^**^	0.737		
GTL	3.497	0.495	0.291^**^	0.379^**^	0.292^**^	0.829	
GSE	3.701	0.425	0.219^*^	0.283^**^	0.303^**^	0.136^*^	0.773

Square root of AVE values for each variable at the diagonal.

* *p < *0.05;

** *p < *0.01;

*** *p < *0.001.

From Table 3, it can be seen that the correlation coefficients between the five latent variables are significant. According to Fornell and Larcker (1981), the arithmetic square root of the average variance extracted (AVE) of the latent variables can be compared with the correlation coefficients between the latent variables to test the discriminant validity between the latent variables. Table 3 shows that the arithmetic square root of AVE values for GHRM practice, green mindfulness, employees’ green behavior, green transformational leadership, and green self-efficacy are 0.735, 0.674, 0.737, 0.829, and 0.773, respectively, which are higher than the threshold of 0.5 set by Fornell and Larcker. The minimum value of the AVE (0.674) square root is greater than the maximum value of the correlation coefficient between the variables (0.639), indicating that the scale has good discriminant validity.

### Hypothesis tests

This study used gender, age, education, years of experience, and job type as control variables. Regarding econometric conventions, four new dummy variables were produced for the job control variables (the original variables were in five categories): administrative jobs, technical jobs, R&D jobs, and production jobs. The base category was other jobs. To test the mediating and moderating effects between the variables, the authors performed regression analysis on the collected data, following Baron and Kenny (1986) steps for testing mediating effects. Hierarchical multiple regressions were used to test the mediating role of environmental commitment and the moderating role of green transformational leadership and green self-efficacy, where M1-M6 denote the six sets of regression analyses performed, respectively. The leftmost column represents the independent variables of the regression model, the top column represents the dependent variables of the regression model, and the results are shown in Table 4.

**Table 4:** Output of the hierarchical linear model.

Variables	GM		GB			
	M1	M2	M3	M4	M5	M6
Control variables						
Gender	−0.024	0.018	−0.015	0.003	−0.005	−0.016
Age	0.018	−0.017	−0.002	−0.015	−0.002	0.004
Education	0.002	0.006	0.012	0.008	−0.012	−0.014
Organizational tenure	0.054	0.007	0.099	0.043	0.01	0.007
Production	−0.045	0.07	0.037	0.066	0.069	0.061
Administrative	0.013	−0.023	0.041	0.025	0.061	0.095
Technical	−0.054	0.031	−0.021	0.011	0.032	0.052
RD	−0.024	−0.028	0.012	0.003	0.007	0.035
GHRM	0.498^***^	0.431^***^	0.548^***^		0.270^***^	
GM				0.780^***^	0.555^***^	0.612^***^
GTL		0.138^***^				0.315^***^
GHRM^*^GTL		0.049^**^				
GSE						0.150^***^
GM^*^GSE						0.095^***^

* *p < *0.05,

** *p < *0.01,

*** *p < *0.001.

As seen in Table 4, Model 3 shows that corporate green HRM practices significantly affect employees’ green behavior (*β* = 0.548, *t* = 13.166, *p* = 0.000); Hypothesis H1 is further verified. Model 1 shows that green HRM practices significantly influence green mindfulness (*β* = 0.500, *t* = 14.287, *p* = 0.000). Model 4 shows that employees’ green mindfulness significantly influences green behavior (*β* = 0.780, *t* = 15.594, *p* = 0.000). Model 6 shows that green transformational leadership significantly influences green behavior (*β* = 0.315, *t* = 7.367, *p* = 0.000), and Hypothesis H2 is further verified. According to Model 5, when green HRM practices and green mindfulness are regressed together, the regression coefficient of green mindfulness is significant (*β* = 0.555, *t* = 8.907, *p* = 0.000). The regression coefficient of green HRM practice decreased (*β* = 0.270, *t* = 5.572, *p* = 0.000), which indicates that there is a partial mediating effect of green mindfulness, and hypothesis H4 is valid. In addition, this study used the bootstrap method to examine the mediating effect of green mindfulness between green HRM and employees’ green behavior by setting the bootstrap random sample 5,000 times. The indirect effect coefficient of green mindfulness is 0.278 with a 95% confidence interval (0.187, 0.389), which does not contain 0. This indicates that the mediating effect of green mindfulness between green HRM and employees’ green behavior is significant. Thus, hypothesis H4 is further supported.

The moderating effects were tested in Models 2 and 6. The coefficient of the interaction term between green HRM practice and green transformational leadership was significant (*β* = 0.049, *t* = 3.246, *p* = 0.001), and the coefficient of the interaction term between green mindfulness and green self-efficacy was significant (*β* = 0.095, *t* = 4.830, *p* = 0.000). The results showed that the confidence interval of the interaction term between corporate green HRM practice and green transformational leadership was (0.085, 0.357). Using the bootstrap method with 5,000 sample resets to test the two moderated mediating effects, the confidence interval of the interaction term between green mindfulness and green self-efficacy was (0.282, 0.780), demonstrating that the moderating mediating effect of green transformational leadership was 0.123, SE = 0.048, CI = (0.035, 0.226) and the moderating mediating effect of green self-efficacy was 0.271, SE = 0.085, CI = (0.117, 0.451). Both hypotheses H3 and H5 were supported.

## Conclusion and discussion

### Research conclusion

Given increasingly severe environmental problems, improving employees’ green behavior has gradually become a topic of concern to all sectors of society. This study considers that green transformational leadership and green human resource management practice are two situational factors to predict employees’ green behavior, integrates these two factors for the first time, and proposes the synergy between them as a factor in predicting and influencing employees’ green behavior. The results show that green transformational leadership and green human resource management practice can predict employees’ green behavior independently and in positive synergistic interaction. Moreover, this paper further reveals the mediating role of green mindfulness and the regulating role of green self-efficacy.

First, this study found that green transformational leadership and green human resource management practice positively interact with employees’ green behavior, which supports the view that “leadership behavior and human resource management practice play a synergistic role.” As a set of hard rules and regulations in the organization, green HRM practices influence the organization or work output in collaboration with the “soft factors” (green transformational leadership) in the organization. When green HRM practices and transformational leaders give consistent and encouraging signals about environmental goals and expectations, these signals reinforce the importance of environmental behaviors in employees’ minds and motivate them to work more environmentally friendly. Second, this study further found that green mindfulness is intermediary in the interaction between green transformational leadership and green human resource management practice. In other words, green leadership and HRM first promote employees’ green mindfulness and then promote employees’ green behaviors. Finally, this study found that green self-efficacy plays a positive moderating role in the influence of the interaction between leadership and HRM practices on employees’ green behavior. When employees have a high level of green self-efficacy, green transformational leadership and green human resource management practice have a more substantial synergistic effect on employees’ green behavior.

### Theoretical contributions

First, this study reveals the positive interaction effect of green transformational leadership and green HRM practices on employees’ green behaviors, which can help the academic community better understand how the two green-related contextual factors jointly influence environmental behaviors. For a long time, scholars have either focused on the impact of green transformational leadership on employees’ green behavior (Graves and Sarkis, 2018) or explored the impact of green HRM practices on employees’ green behavior (Zhou and Zhang, 2018) but have neglected the joint role of these two important contextual factors. By revealing the synergistic mechanism of green management on employees’ green behavior, this study can bridge the gap between the two fields of green leadership and green HRM and contribute to a comprehensive understanding of the mechanisms that induce green behavior in the workplace.

Second, based on resource conservation theory, this study finds that green mindfulness plays an intermediary role in the interaction between green transformational leadership and green human resource management practice, thus revealing how the two jointly affect employees’ green behavior. Although existing studies have used organizational recognition (Shen et al., 2018), a green psychological atmosphere (Dumont et al., 2017), work motivation (Longoni et al., 2018; Zhou and Zhang, 2018), environmental knowledge (Saeed et al., 2019; Fawehinmi et al., 2020) and other variables to discuss the impact of green human resource management on employees’ green behavior, the existing research has ignored the role of green mindfulness as an important variable in explaining the impact of green management on employees’ green output (Luu, 2019; Shafaei et al., 2020). From the perspective of resource conservation theory, this study examines the internal psychological mechanism of how work resources (green human resource management practice) are transformed into employees’ green output (green behavior). Consistent with previous research (Dumont et al., 2017; Luu, 2019; Zhang et al., 2019), green human resource management indirectly affects employees’ green behavior through the “bridge” of green mindfulness, which further enriches and develops the application of green mindfulness in the field of green human resource management.

Third, this study found that green self-efficacy has a significant positive moderating effect on the relationship between green human resource management and employees’ green behavior. Previous studies have shown that the interaction between green human resource management and individual factors (such as environmental values and environmental knowledge) has a positive impact on employees’ green output (Dumont et al., 2017; Saeed et al., 2019; Hameed et al., 2020). This study explores the effect of green self-efficacy on the differentiation of green human resource management on employees’ green behavior. It is found that green self-efficacy is an important contextual variable affecting employees’ green behavior. When employees have a high level of green self-efficacy, green human resource management has a stronger effect on green behavior. This study enriches the boundary conditions of green human resource management and further clarifies the conditions under which it is more conducive to promoting employees’ green behavior, which has important theoretical significance for enterprises to implement green management.

### Practical implications

First, companies should be aware of the key role that green HRM practices play in improving individual environmental performance and subsequent organizational effectiveness. At the managerial level, companies should establish relevant rules and regulations based on green HRM practices, create suitable conditions to develop an atmosphere of environmental responsibility, enhance employees’ perception of corporate environmental responsibility, and reduce employees’ negative emotions in the workplace while making it easier for them to accept the environmental values advocated by the organization, which helps to generate green behaviors. The HR departments of enterprises can assess the green mindfulness and green self-efficacy of job applicants through relevant tests in the interview process to recruit and select employees with environmental values.

Second, the role of leaders is crucial in the process of enhancing employees’ green mindfulness and stimulating their green behaviors because leadership attitudes toward environmental protection are an important factor in promoting employees’ green ideas and behaviors. Green transformational leaders attach importance to sustainable development, take sustainable development goals as their guide, and have strong environmental consciousness. Continuously promoting and advocating for a company’s environmental protection policies is conducive to enhancing the effectiveness of green HRM practices and promoting the green behavior of employees.

Third, because individuals with high green mindfulness tend to choose green behavior (Barbaro and Pickett, 2016), organizations should attach great importance to green mindfulness. For example, managers should incorporate green mindfulness into the evaluation system for recruitment. In the process of making a green training program, a green mindfulness course should be introduced, and a green knowledge and information sharing platform should be constructed to increase employees’ concentration and awareness of their current experience. Opportunities for employees to participate in the formulation of green initiatives should be provided along with various forms of green learning research conferences or forums, and encouragement for employees to put forward green suggestions and problem-solving methods to cultivate green mindfulness.

Finally, in the process of stimulating employees’ green behavior, enterprises should not only train green transformational leaders or implement green HRM policies but also consider both aspects (hard and soft) as much as possible to more effectively promote employees’ green behavior and the sustainable development of the organization. Specifically, companies need to train leaders in green leadership, improve their knowledge and skills, teach them how to set green examples, motivate employees to be green, and support them in green practices. However, there are limitations to relying solely on the subjective norms of leaders to manage. For example, it is not easy to ensure that every employee will be convinced of the values communicated by the leader. For this reason, companies should also develop a standardized system for green management, such as incorporating environmental issues into the major modules of human resource management, cultivating, motivating and evaluating employees’ green performance, and providing institutional safeguards for leaders’ green management behavior. By combining green transformational leadership and green HRM practices, we can take advantage of each other’s strengths and complement each other’s weaknesses to promote green employee management.

### Limitations and future research

While this study validates the target findings, it also has certain limitations that need to be addressed in future research. First, this theoretical study only examines the synergistic mechanism between employees’ perceived green human resource management practice and green transformational leadership as an influence on green behavior at the individual level. Previous studies have tested the relationship between human resource management practice (Wang and Xu, 2017), green mindfulness (Yu and Zellmer-Bruhn, 2018), responsible leadership (Huang and Xu, 2021) and green behavior at the team level. Future studies can explore the influence of green human resource management practice and green transformational leadership on team green behavior and its mechanism from the team level. Second, this study focuses on the mediating role of green mindfulness, which is only one of the synergistic outputs of green HRM and green transformational leadership. Future research can explore the synergistic mechanism of green human resource management practice and green transformational leadership by using variables such as green job remodeling (Luu, 2019), green psychological atmosphere (Dumont et al., 2017), environmental knowledge (Fawehinmi et al., 2020) and green psychological capital (Saeed et al., 2019). Third, although it is essential to promote employees’ green behaviors to improve the environmental performance of enterprises, it is necessary to further explore whether green HRM practices can improve the environmental performance of enterprises while taking into account economic performance. Suppose this reduces the economic efficiency of enterprises. In that case, it is necessary to integrate the theoretical basis and research findings of related disciplines to achieve an effective balance between environmental and financial performance.

## Data availability statement

The original contributions presented in the study are included in the article/Supplementary material; further inquiries can be directed to the corresponding author.

## Ethics statement

Ethical review and approval was not required for the study on human participants in accordance with the local legislation and institutional requirements. The patients/participants provided their written informed consent to participate in this study.

## Author contributions

ZW collected and analyzed the data. TC framed the introduction and discussion. All authors contributed to the article and approved the submitted version.

## Funding

This work was supported by the National Social Science Funds of China under Grant 20BGL192 and 21BGL248, the General Project of Humanities and Social Sciences Research in Universities of Henan Province under Grant 2023-ZZJH-027, the Teacher Development Research and Practice Project of Henan Polytechnic Institute under Grant JSFZ202202, and the Vocational Education Research Project of Henan Polytechnic Institute under Grant 2022ZJYJ12. The funders had no role in the study design, data collection and analysis, decision to publish, or preparation of the manuscript.

## Conflict of interest

The authors declare that the research was conducted in the absence of any commercial or financial relationships that could be construed as a potential conflict of interest.

## Publisher’s note

All claims expressed in this article are solely those of the authors and do not necessarily represent those of their affiliated organizations, or those of the publisher, the editors and the reviewers. Any product that may be evaluated in this article, or claim that may be made by its manufacturer, is not guaranteed or endorsed by the publisher.

## Supplementary material

The Supplementary material for this article can be found online at: https://www.frontiersin.org/articles/10.3389/fpsyg.2022.906869/full#supplementary-material
