# Supplementary material for: How to facilitate employees’ green behavior? The joint role of green human resource management practice and green transformational leadership
Source: Front Psychol. 2022 Aug 11;13:906869. doi: 10.3389/fpsyg.2022.906869 (PMC9403470; doi:10.3389/fpsyg.2022.906869)
Supplement: Supplementary file 1 [file Data_Sheet_1.docx]

Supplementary Material

# Supplementary Tables

**Table 1.** Variable measurement scales

| **Latent Variables** | **Observed**  **Variables** | **Measure Items** |
| --- | --- | --- |
| **GHRM** | A11 | My company sets green goals for its employees. |
|  | A12 | My company provides employees with green training to promote green values. |
|  | A13 | My company provides employees with green training to develop employees’ knowledge and skills required for green management. |
|  | A14 | My company considers employees’ workplace green behavior in performance appraisals. |
|  | A15 | My company relates employees’ workplace green behaviors to rewards and compensation. |
|  | A16 | My company considers employees’ workplace green behaviors in promotion. |
| **GM** | B21 | I feel free to discuss environmental issues and problems; |
|  | B22 | I have been encouraged to express different views with respect to environmental issues and problems. |
|  | B23 | I pay attention to what is happening if unexpected environmental issues and problems arise. |
|  | B24 | I have been inclined to report environmental information and knowledge that have significant consequences. |
|  | B25 | I have been rewarded if I share and announce new environmental information and knowledge. |
|  | B26 | I know what is readily available for consultation if unexpected environmental issues and problems arise. |
| **GTL** | C31 | My leader inspires us with the environmental plans. |
|  | C32 | My leader provides a clear environmental vision for us to follow. |
|  | C33 | My leader gets us to work together for the same environmental goals. |
|  | C34 | My leader states that he is committed to improving the environmental performance of the organization. |
|  | C35 | My leader states many times that he values the natural environment. |
|  | C36 | My leader motivates me to work in an environmentally friendly way. |
| **GSE** | D41 | I feel I can succeed in accomplishing environmental ideas. |
|  | D42 | I can achieve most of environmental goals. |
|  | D43 | I feel competent to deal effectively with environmental tasks. |
|  | D44 | I can perform effectively on environmental missions. |
|  | D45 | I can overcome environmental problems. |
|  | D46 | I could find out creative solutions to environmental problems. |
| **GB** | E51 | At work, I find ways of working that are better for the environment. |
|  | E52 | At work, I apply new ideas for reducing our impact on the environment. |
|  | E53 | At work, I help create green processes and products. |
|  | E54 | At work, I question practices that are likely to hurt the environment. |
|  | E55 | At work, I recycle and reuse materials. |
|  | E56 | At work, I try to reduce my energy use. |
|  | E57 | At work, I join in environmental activities that are not required by my job. |

**Table 2.** Basic characteristics of the sample

| Demographic Information | | Respondents (N = 296) | Percentage (%) |
| --- | --- | --- | --- |
| **Gender** | Male | 167 | 56.42 |
|  | Female | 129 | 43.58 |
| **Age** | Less than 30 years | 125 | 42.23 |
|  | 31-40years | 113 | 38.18 |
|  | 41-50years | 41 | 13.85 |
|  | 51 and over | 17 | 5.74 |
| **Education** | High school and above | 57 | 19.26 |
|  | Associate Degree | 93 | 31.42 |
|  | Bachelor's degree | 114 | 38.51 |
|  | Master's degree or above | 32 | 10.81 |
| **Organizational Tenure** | 1–3 years | 136 | 45.95 |
|  | 4–6 years | 78 | 26.35 |
|  | 7–9 years | 44 | 14.86 |
|  | 10 years and above | 38 | 12.84 |
| **Positions** | Production | 114 | 38.51 |
|  | Administrative | 67 | 22.64 |
|  | Technical | 55 | 18.58 |
|  | R&D | 20 | 6.76 |
|  | Other | 40 | 13.51 |

**Table 3.** Scale reliability and factor analysis results

| **Latent Variables** | **Observed**  **Variables** | **Factor**  **Loading** | **CR** | **AVE** | **Cronbach α** |
| --- | --- | --- | --- | --- | --- |
| **GHRM** | A11 | 0.546 | 0.868 | 0.540 | 0.903 |
|  | A12 | 0.527 |  |  |  |
|  | A13 | 0.985 |  |  |  |
|  | A14 | 0.975 |  |  |  |
|  | A15 | 0.613 |  |  |  |
|  | A16 | 0.608 |  |  |  |
| **GM** | B21 | 0.22 | 0.821 | 0.455 | 0.823 |
|  | B22 | 0.754 |  |  |  |
|  | B23 | 0.651 |  |  |  |
|  | B24 | 0.771 |  |  |  |
|  | B25 | 0.749 |  |  |  |
|  | B26 | 0.73 |  |  |  |
| **GTL** | C31 | 0.971 | 0.929 | 0.688 | 0.921 |
|  | C32 | 0.911 |  |  |  |
|  | C33 | 0.76 |  |  |  |
|  | C34 | 0.677 |  |  |  |
|  | C35 | 0.908 |  |  |  |
|  | C36 | 0.703 |  |  |  |
| **GSE** | D41 | 0.446 | 0.893 | 0.597 | 0.900 |
|  | D42 | 0.832 |  |  |  |
|  | D43 | 0.882 |  |  |  |
|  | D44 | 1.003 |  |  |  |
|  | D45 | 0.545 |  |  |  |
|  | D46 | 0.785 |  |  |  |
| **GB** | E51 | 0.561 | 0.888 | 0.543 | 0.890 |
|  | E52 | 0.929 |  |  |  |
|  | E53 | 0.817 |  |  |  |
|  | E54 | 0.722 |  |  |  |
|  | E55 | 0.462 |  |  |  |
|  | E56 | 0.688 |  |  |  |
|  | E57 | 0.863 |  |  |  |

**Table 5.** Mean value, standard deviation and correlation coefficient of five variables (N=296)

| **Variable** | **Mean** | **SD** | **GM** | **GB** | **GTL** | **GSE** |  |
| --- | --- | --- | --- | --- | --- | --- | --- |
| GHRM | 3.421 | 0.444 | 0.735 |  |  |  |  |
| GM | 3.292 | 0.345 | 0.639** | 0.674 |  |  |  |
| GB | 3.328 | 0.396 | 0.608** | 0.677** | 0.737 |  |  |
| GTL | 3.497 | 0.495 | 0.291** | 0.379** | 0.292** | 0.829 |  |
| GSE | 3.701 | 0.425 | 0.219* | 0.283** | 0.303** | 0.136* | 0.773 |

Note: *p < 0.05, **p < 0.01, ***p < 0.001, square root of AVE values for each variable at the diagonal.

**Table 6. Results of bootstrap test**

|  | coeffect | SE | t | P | LLCI | ULCI |
| --- | --- | --- | --- | --- | --- | --- |
| Int1(GHRM*GTL) | 0.222 | 0.069 | 3.246 | 0.0013 | 0.085 | 0.357 |
| Int2(GM*GSE) | 0.540 | 0.131 | 4.116 | 0.0001 | 0.282 | 0.780 |
| GTL(moderated mediation) | 0.123 | 0.048 |  |  | 0.035 | 0.226 |
| GSE(moderated mediation) | 0.271 | 0.085 |  |  | 0.117 | 0.451 |
